# Operated case of adolescent idiopathic scoliosis at dorsal spine with recurrence of deformity: an unusual presentation

**DOI:** 10.11604/pamj.2023.45.182.40881

**Published:** 2023-08-25

**Authors:** Hardik Rasiklal Patel, Sohael Khan

**Affiliations:** 1 Department of Orthopedics, Datta Meghe Institute of Medical Sciences (DU), Sawangi, Wardha, Maharashtra, India

**Keywords:** Scoliosis, recurrence of deformity, adolescent idiopathic scoliosis

## Image in medicine

Adolescent idiopathic scoliosis is a coronal plane spinal deformity which most commonly presents in adolescent girls from ages 10 to 18 (A). Diagnosis is made with full-length standing posteroanterior (PA) and lateral spine radiographs. Treatment can be observation, bracing, or surgical management depending on the skeletal maturity of the patient, magnitude of deformity, and curve progression. In scoliosis cases, surgical intervention is typically considered when the curvature progresses to a certain degree or when it causes significant pain, respiratory problems, or other complications (B, C). The goal of scoliosis surgery is to correct the curvature and stabilize the spine. However, despite surgical intervention, there is a small risk of recurrence or progression of the deformity. Factors that may increase the risk of recurrence include inadequate surgical correction, incomplete fusion of the spinal segments, failure of implants or instrumentation, and continued growth of the spine in skeletally immature individuals. Here, we report a rare presentation of operated case of adolescent idiopathic scoliosis at dorsal spine with recurrence of deformity (D). Recurrence of deformity is very uncommon (<0.5 %). A 22-year-old female patient was brought to the orthopedics outpatient department (OPD) with complaints of pain and deformity in his upper back region since 5 years which were increasing over the period of time (E). On examination, there was scar of previous surgery present over midline approximately 15 cm in length at dorso lumbar level, recurrence of deformity over right side convex curve and no neurodeficit present and power in bilateral upper limb and lower limb v/v.

**Figure 1:**
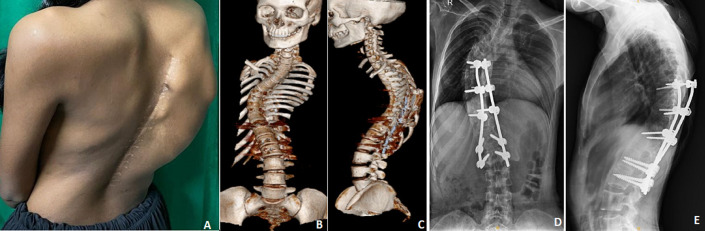
A) clinical photographs of old operated case of adolescent idiopathic scoliosis at dorsal spine with recurrence of deformity; B,C) Computed tomography scan image showing operated case of scoliosis with implant in situ with apex of deformity towards right side and recurrence of deformity start from proximal end of implant; D) anteroposterior view of X-ray image showing operated case of scoliosis with implant in situ with apex of deformity towards right side and concave portion towards left side and recurrence of deformity start from D9 vertebrae; E) lateral view of X-ray image showing operated case of scoliosis with implant in situ with apex of deformity towards right side and concave portion towards left side and recurrence of deformity start from D9 vertebrae

